# An Updated Meta-Analysis of the Relationship Between *Helicobacter pylori* Infection and the Risk of Coronary Heart Disease

**DOI:** 10.3389/fcvm.2022.794445

**Published:** 2022-04-29

**Authors:** Ling Tong, Bei-Bei Wang, Fei-Hong Li, Shu-Ping Lv, Fei-Fei Pan, Xin-Jiang Dong

**Affiliations:** ^1^Department of Cardiology, Shanxi Provincial People’s Hospital, Taiyuan, China; ^2^Department of Cardiology, The First People’s Hospital of Jinzhong, Jinzhong, China; ^3^Department of Cardiology, Yantai Yeda Hospital, Yantai, China; ^4^Department of Cardiology, Shanxi Bethune Hospital, Shanxi Academy of Medical Sciences, Taiyuan, China; ^5^Department of Cardiology, The First Hospital of Shanxi Medical University, Taiyuan, China; ^6^Department of Cardiology, Shanxi Cardiovascular Hospital, Taiyuan, China

**Keywords:** coronary heart disease, *Helicobacter pylori*, anti-*H. pylori* IgG test, anti-CagA test, *H. pylori* stool antigen test, *H. pylori* histological staining test, systematic review, meta-analysis

## Abstract

**Background:**

Coronary heart disease (CHD) is one of the leading causes of mortality in the world. Although the traditional risk factors for CHD have been identified, it seems that there are still many CHD cases without these factors. Previous studies have hypothesized that *Helicobacter pylori* (*H. pylori*) infection was associated with the risk of CHD.

**Objective:**

The association between *H. pylori* infection and the risk of CHD was studied using a systematic evaluation and meta-analysis method.

**Methods:**

In order to find relevant studies, four electronic databases were systematically searched until August 2021. According to the inclusion and exclusion criteria, studies were screened and data were extracted. Under the random-effects or the fixed-effects model, the odds ratio (OR) and 95% confidence interval (95% CI) were combined. All analyses were conducted using Review Manager software (RevMan 5.4).

**Results:**

Among the included studies, 2 studies were analyzed for *H. pylori* stool antigen test, 2 studies were analyzed for *H. pylori* histological staining test, 13 studies were analyzed for the anti-CagA test, and 38 studies were analyzed for the anti-*H. pylori* IgG test. The pooled results revealed that positive anti-*H. pylori* IgG was significantly associated with an increased risk of CHD (OR, 1.58; 95% CI: 1.34–1.87). Similarly, positive anti-CagA, positive *H. pylori* stool antigen, and positive *H. pylori* histological staining were significantly associated with the development of CHD with (OR: 1.33, 95% CI: 1.16–1.53), (OR: 3.50, 95% CI: 1.60–7.66), and (OR: 1.78, 95% CI: 1.12–2.83), respectively.

**Conclusion:**

This meta-analysis showed that *H. pylori* infection increased the risk of CHD. However, more studies are needed to further investigate whether early eradication of *H. pylori* may reduce the morbidity of CHD.

## Introduction

*Helicobacter pylori* (*H. pylori*), a gram-negative bacterium, is one of the common infections in human. More than the half of population in the world suffers from the infection (1). *H. pylori* infection causes a wide range of gastrointestinal diseases including chronic gastritis, gastric cancer, and duodenal ulcer (2, 3). Moreover, researchers have also recently found that *H. pylori* infection was closely related to atherosclerotic cardiovascular diseases, including coronary heart disease (CHD), peripheral arterial disease, and stroke (4–7).

CHD is the most common type of organ disease caused by atherosclerosis, the leading cause of mortality in many countries (8). The etiology and pathogenesis of CHD have not been fully understood until now. The classical risk factors, including diabetes, hypertension, obesity, smoking, dyslipidemia, socioeconomic status, and family history, cannot fully explain all causes (9). Chronic inflammation caused by chronic infection, such as *H. pylori* infection, plays an important role in the pathogenesis of CHD. Some studies have shown that *H. pylori* infection increased the risk of CHD (10, 11). At the same time, other studies have shown that *H. pylori* infection was not closely related to CHD (12, 13). Previous meta-analyses have also provided evidence for or against the relationship between *H. pylori* infection and the risk of CHD (14, 15).

Previous studies have been controversial, even from earlier published meta-analyses with no clear final conclusions. Therefore, we conducted a large-scale systematic review and meta-analysis to establish specific evidence about the relationship between *H. pylori* infection and the risk of CHD.

## Materials and Methods

This meta-analysis strictly followed the recommendations of the systematic review and meta-analysis (PRISMA) list (16) of the preferred reporting items.

### Literature Search

We searched four electronic databases including Web of Science, Embase, PubMed, and Cochrane Library until August 2021. We used the following search string: “ischemic heart disease (IHD)” OR “coronary heart disease (CHD)” OR “coronary artery disease (CAD)” OR “coronary atherosclerosis” OR “angina” OR “unstable angina (UA)” OR “acute myocardial infarction (AMI)” OR “Acute coronary syndrome (ACS)” OR “myocardial infarction (MI)” OR “atheroma” AND “*Helicobacter*” OR “*Helicobacter pylori*” OR “*Campylobacter pylori*” OR “*H. pylori.*” The references in the included studies were checked, and suitable studies were identified.

### Literature Selection and Data Extraction

Eligible studies that reported the relationship between *H. pylori* infection and the risk of CHD were included in this meta-analysis. In addition to studies with unreliable data, we excluded abstract-only articles, book chapters, conference papers, theses, reviews, letters, editorials, and posters. There were no restrictions for included studies on publication year, language, place, or demographics of patients. Any discrepancy in the screening step was agreed by two reviewers. If necessary, a third reviewer was consulted. Then, the full-text screening was carried out to identify the related studies for data extraction. The extracted data included the following: the first author, publication year, category of CHD, country, settings, study design, sample size, agent, and adjustment status.

### Quality and Assessment

All studies were evaluated using the modified Newcastle-Ottawa Scale (NOS). This scoring system assessed studies according to the comparability of groups, patient selection, and assessment outcomes. When an article scored >7 points in this scoring system, it was considered a high-quality article.

### Statistical Analysis

The most adjusted hazard ratio (HR) or odds ratio (OR) with 95% confidence interval (CI) was extracted and combined into an equivalent measure which is expressed in the form of combined OR. The original data was extracted and used to calculate the original OR when a study did not contain the most adjusted HR or OR with 95% CI. Heterogeneity among studies was assessed by using the *I*-statistic (*I*^2^) test (17). When there was substantial heterogeneity (*I*^2^ > 50%), the random-effects model was used. Otherwise, the fixed-effects model was selected (18). In order to test the robustness of the results, the sensitivity analysis was evaluated by excluding one study at a time (19). In addition, several subgroup meta-analyses were performed based on study design, setting, adjustment status, quality assessment score, category of CHD, and country. All analyses were conducted using Review Manager (RevMan 5.4), and a *p*-value of less than 0.05 was regarded as statistically significant.

## Results

### Search Results

**FIGURE 1:**
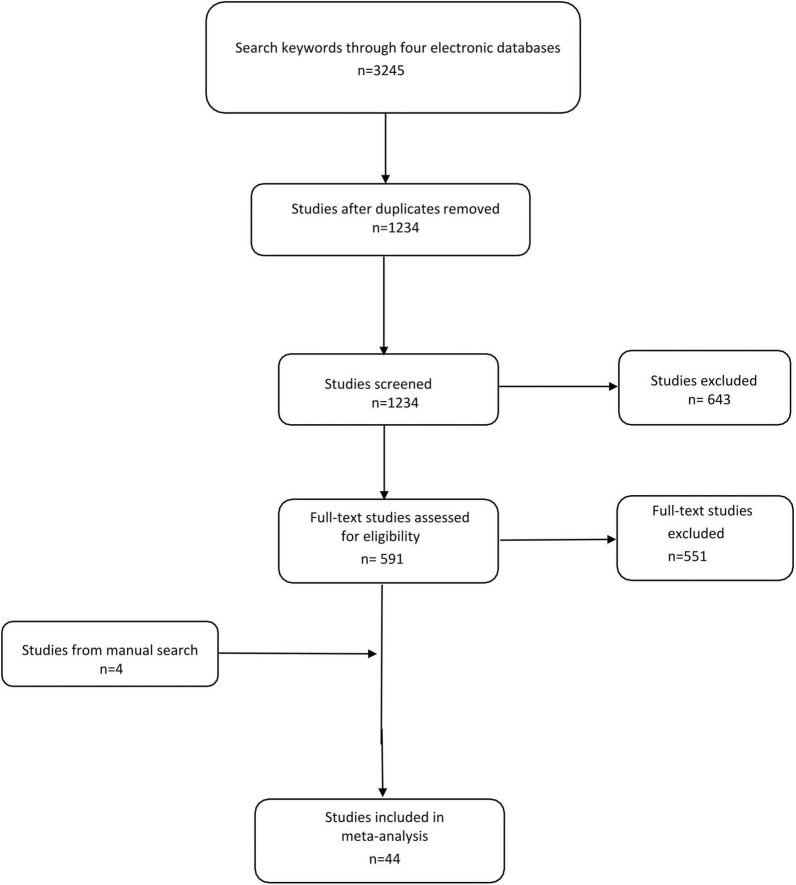
Selection process for studies included in the meta-analysis.

Using predefined keywords, 3,245 studies were identified from four electronic databases. After excluding duplicate studies, 1,234 studies were included and screened, of which 643 were deleted by title and abstract. We screened the remaining 591 studies and excluded 551 studies according to the exclusion criteria. In addition, the manual search yielded 4 other studies. We then extracted useful data from 44 qualified studies (20–63) (Figure 1).

### Characteristics of the Included Studies

Among the included studies, 13 studies were prospective studies with a total sample size of 16,236 participants. Thirty-one studies were cross-sectional studies with 14,689 participants. Included studies were published between 1995 and 2017. The number of included studies per type of CHD were as follows: 19 studies on CHD, 12 studies on AMI, 10 studies on MI, 2 studies on IHD, and 1 study on ACS. Ten studies were conducted in the United Kingdom, 5 in Iran, 4 in the United States, 4 in Italy, 4 in Japan, 3 in India, 3 in Turkey, 2 in China, 2 in South Korea, 1 in New Zealand, 1 in the Netherlands, 1 in Sweden, 1 in Germany, 1 in Greece, 1 in Pakistan, and 1 in Croatia. We found 27 studies that were conducted in hospital-based settings and 17 studies conducted in a community-based setting. *H. pylori* detection method included anti-*H. pylori* IgG, anti-CagA, *H. pylori* stool antigen, and *H. pylori* histological staining. According to the adjustment status, there were 17 adjusted studies and 27 unadjusted studies. Moreover, the included studies were divided into 21 studies with ≥7 points and 23 studies with <7 points according to the quality score (Table 1).

**TABLE 1:** Description of included studies.

References	CHD type	Country	Setting	Sample size^a^	Study design	Agent	Adjustment state	Quality score
Patel et al. (20)	CHD	United Kingdom	Community	26/341	CS	1	N	8
Whincup et al. (21)	MI	United Kingdom	Community	135/136	PS	1	Y	7
Rathbone et al. (22)	AMI	United Kingdom	Hospital	342/236	CS	1	Y	7
Folsom et al. (23)	CHD	United States	Community	217/498	PS	1	Y	8
Pellicano et al. (24)	AMI	Italy	Hospital	44/310	CS	1	N	6
Danesh et al. (25)	MI	United Kingdom	Community	1,122/1,122	CS	1	Y	7
Galante et al. (26)	MI	Italy	Hospital	63/61	CS	1	N	6
Kahan et al. (27)	AMI	Sweden	Hospital	100/100	CS	1	Y	6
Gunn et al. (28)	AMI	United Kingdom	Hospital	342/214	CS	1	N	7
Ridker et al. (29)	MI	United States	Community	445/445	PS	1	N	8
Kinjo et al. (30)	AMI	Japan	Hospital	618/967	CS	1	Y	7
Fraser et al. (31)	MI	New Zealand	Community	341/831	CS	1	Y	6
Ozdogru et al. (32)	MI	Turkey	Hospital	353/163	CS	1	N	5
Nikolopoulou et al. (33)	AMI	Greece	Hospital	138/49	CS	1	N	6
Jafarzadeh et al. (34)	CHD	Iran	Hospital	120/60	CS	1, 2	N	6
Guan et al. (35)	AMI	China	Community	150/102	CS	1	N	5
Nakić et al. (36)	AMI	Croatia	Hospital	93/100	PS	1	N	6
Khodaii et al. (37)	AMI	Iran	Hospital	500/500	CS	1, 2	N	5
Padmavati et al. (38)	CHD	India	Hospital	108/100	CS	1	N	6
Schöttker et al. (39)	MI	Germany	Community	8,482/154	PS	1,2	Y	7
Ikeda et al. (40)	MI	Japan	Community	106/212	PS	1, 2	N	8
Sunanda et al. (41)	AMI	India	Community	261/261	CS	1	N	6
Witherell et al. (42)	MI	United States	Community	121/201	PS	1	N	6
Singh et al. (43)	CHD	United Kingdom	Hospital	201/414	PS	2	Y	7
Ossewaarde et al. (44)	CHD	Netherlands	Community	54/108	PS	1	N	6
Whincup et al. (45)	CHD	United Kingdom	Community	505/1,025	PS	1, 2	Y	7
Wald et al. (46)	CHD	United Kingdom	Community	648/1,296	PS	1	N	8
Stone et al. (47)	CHD	United Kingdom	Community	172/205	PS	1, 2	N	8
Danesh et al. (48)	CHD	United Kingdom	Community	288/704	CS	1	Y	7
Azarkar et al. (49)	MI	Iran	Hospital	73/78	CS	1	N	6
Khurshid et al. (50)	CHD	United States	Hospital	58/121	CS	1	Y	7
Pasceri et al. (51)	IHD	Iran	Hospital	88/88	CS	1, 2	Y	8
Bonaventura et al. (52)	IHD	Iran	Hospital	58/52	CS	2	N	6
Lenzi et al. (53)	CHD	Italy	Hospital	80/160	CS	2	N	5
Zodpey et al. (54)	AMI	India	Hospital	265/265	CS	1	Y	6
Aceti et al. (55)	CHD	Italy	Hospital	40/40	CS	2, 3	N	7
Tsai and Huang (56)	CHD	China	Hospital	165/127	CS	1	Y	5
Jin et al. (57)	CHD	South Korea	Hospital	175/88	PS	3	N	6
Miyazaki et al. (58)	ACS	Japan	Hospital	33/66	CS	1, 2	Y	7
Adiloglu et al. (59)	CHD	Turkey	Hospital	38/12	CS	4	N	5
Adiloglu et al. (60)	CHD	Turkey	Hospital	88/91	CS	1, 2	N	6
Lee et al. (61)	CHD	South Korea	Hospital	54/40	CS	4	N	6
Lin et al. (62)	CHD	Japan	Community	627/627	CS	1	Y	7
Bai and Hashmi (63)	AMI	Pakistan	Hospital	109/109	CS	1	N	7

*CHD, coronary heart disease; ACS, acute coronary syndrome; AMI, acute myocardial infarction; IHD, ischemic heart disease; MI, myocardial infarction; CS, cross-sectional study; PS, prospective study; Y, adjusted articles; N, unadjusted articles.*

*^a^No. of participants with CHD/no. of participants without CHD. 1, detection of H. pylori IgG by ELISA or chemiluminescence; 2, H. pylori CagA positive strains by immunoenzymatic method; 3, H. pylori stool antigen by enzyme immunoassay; 4, H. pylori histological staining by Warthin–Starry silver stain. Diagnosis of CHD, IHD, ACS, AMI and MI by medical history, symptoms, signs, ECG, cardiac ultrasound and coronary angiography, etc.*

### Main Results

We revealed the relationship between the risk of CHD and *H. pylori* infection by using different *H. pylori* detection methods.

### Anti-*Helicobacter pylori* IgG Test and Coronary Heart Disease

**FIGURE 2:**
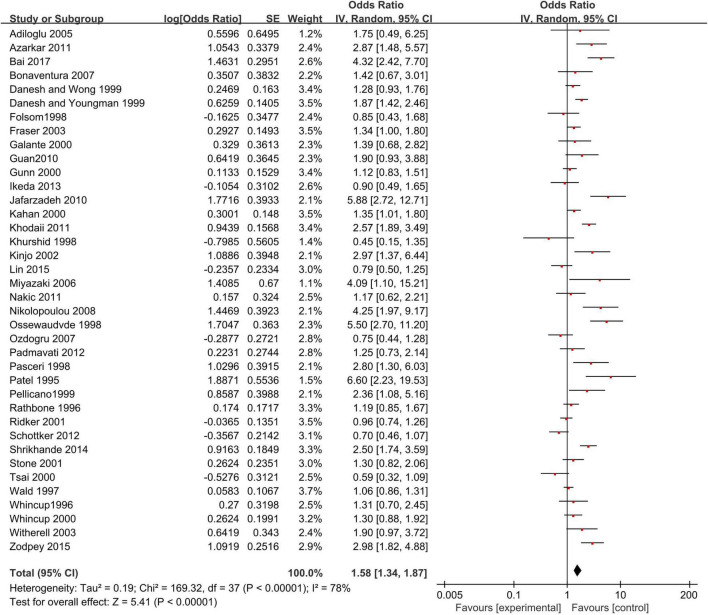
Relationship anti-*H. pylori* IgG test and coronary heart disease [CHD; studies reporting OR + studies reporting hazard ratio (HR)].

**FIGURE 3:**
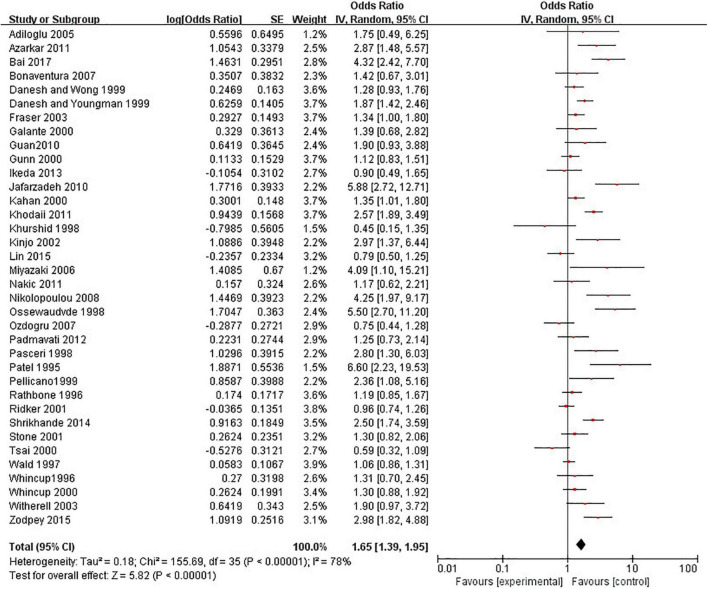
Relationship anti-*H. pylori* IgG test and CHD in studies reporting odds ratio (OR).

**FIGURE 4:**
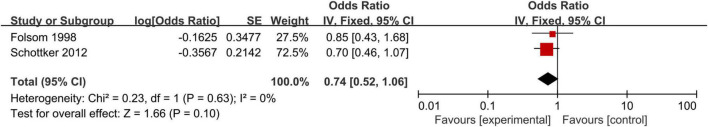
Relationship anti-*H. pylori* IgG test and CHD in studies reporting HR.

A meat-analysis of 38 studies, of which 2 studies used HR and 36 studies used OR, indicated a statistically significant relationship between the risk of CHD and positive anti-*H. pylori* IgG (OR, 1.58; 95% CI: 1.34–1.87; Figure 2). In addition, the meta-analysis of studies reporting OR also showed a statistically significant relationship (OR, 1.65; 95% CI: 1.39–1.95; Figure 3). However, the meta-analysis of studies reporting HR showed a statistically non-significant relationship (HR 0.74; 95% CI: 0.52–1.06; Figure 4). Subgroup analyses based on study design, setting, adjustment, quality assessment score, the category of CHD, and country are presented in Table 2. A leave-one-out sensitivity analysis showed robust results, and none of the studies had a significant impact on the pooled results.

**TABLE 2:** Subgroup analyses about between anti-*H. pylori* IgG and CHD.

Category of subgroups	Subgroups	Number of studies	OR	95% CI	*I* ^2^	*P-*value
Study design	PS	11	1.21	0.95–1.53	67	0.12
	CS	27	1.78	1.45–2.18	77	<0.00001
Setting	Hospital	21	1.79	1.38–2.32	77	<0.0001
	Community	17	1.39	1.12–1.72	77	0.002
Adjustment state	Adjusted	16	1.31	1.05–1.62	72	0.02
	Not adjusted	22	1.86	1.45–2.39	81	<0.00001
Quality assessment	≥7	19	1.33	1.09–1.63	74	0.006
	<7	19	1.88	1.47–2.42	75	<0.00001
Category of CHD	MI	10	1.24	0.96–1.62	75	0.010
	AMI	12	2.06	1.56–2.71	78	<0.00001
	ACS	1	4.09	1.10–15.21	–	0.04
	CHD	13	1.41	1.02–1.93	79	0.03
	IHD	2	1.98	1.16–3.39	35	0.01
Country	United Kingdom	9	1.34	1.11–1.62	60	0.003
	United States	4	0.99	0.79–1.25	48	0.96
	Iran	5	2.68	2.12–3.39	42	<0.00001
	India	3	2.15	1.35–3.43	68	0.001
	Japan	4	1.49	0.72–3.09	76	0.28
	China	2	1.04	0.33–3.28	83	0.94
	Turkey	2	0.85	0.52–1.39	31	0.52
	Italy	2	1.76	1.04–2.98	0	0.03
	New Zealand	1	1.34	1.00–1.80	–	0.05
	Netherlands	1	5.50	2.70–11.20	–	<0.00001
	Sweden	1	1.35	1.01–1.80	–	0.04
	Germany	1	0.70	0.46–1.07	–	0.10
	Croatia	1	1.17	0.62–2.21	–	0.63
	Greece	1	4.25	1.97–9.17	–	0.0002
	Pakistan	1	4.32	2.42–7.70	–	<0.00001

*CHD, coronary heart disease; ACS, acute coronary syndrome; AMI, acute myocardial infarction; IHD, ischemic heart disease; MI, myocardial infarction; CS, cross-sectional study; PS, prospective study.*

### Anti-CagA Test and Coronary Heart Disease

**FIGURE 5:**
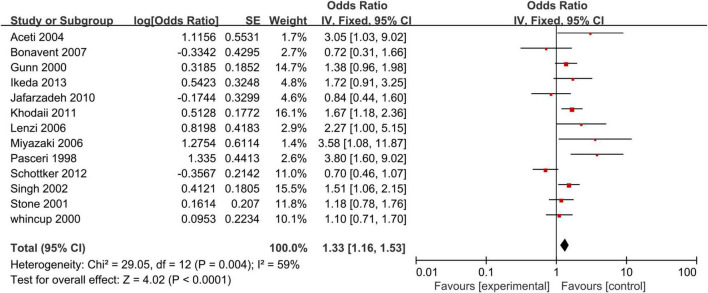
Relationship anti-CagA test and CHD.

Our analysis of 13 studies showed a significant correlation between the risk of CHD and positive anti-CagA (OR, 1.33; 95% CI: 1.16–1.53; Figure 5). One study was reported using an adjusted result. Subgroup analyses based on study design, setting, adjustment, quality assessment score, the category of CHD, and country are presented in Table 3. A leave-one-out sensitivity analysis also indicated that the results are robust, and none of the studies had a significant influence on the overall results.

**TABLE 3:** Subgroup analyses about between anti-CagA test and CHD.

Category of subgroups	Subgroups	Number of studies	OR	95% CI	*I* ^2^	*P-*value
Study design	PS	5	1.16	0.96–1.40	57	0.13
	CS	8	1.56	1.27–1.91	55	<0.0001
Setting	Hospital	10	1.47	1.25–1.73	49	<0.00001
	Community	3	1.02	0.78–1.33	67	0.88
Adjustment	Adjusted	6	1.35	1.12–1.62	75	0.002
	Not adjusted	7	1.31	1.06–1.62	33	0.01
Quality assessment	≥7	9	1.31	1.11–1.54	9	0.001
	<7	4	1.40	1.07–1.84	4	0.01
Category of CHD	MI	2	0.92	0.65–1.31	2	0.64
	AMI	2	0	1.18–1.96	2	0.001
	ACS	1	3.58	1.04–11.87	NG	0.04
	CHD	8	1.34	1.10–1.63	54	0.003
Country	United Kingdom	4	1.31	1.08–1.58	0	0.007
	Italy	2	2.53	1.31–4.86	0	0.005
	Iran	4	1.47	1.12–1.93	72	0.006
	Japan	2	2.02	1.15–3.55	11	0.01
	Germany	1	0.70	0.46–1.07	NG	0.10

*CHD, coronary heart disease; ACS, acute coronary syndrome; AMI, acute myocardial infarction; IHD, ischemic heart disease; MI, myocardial infarction; CS, cross-sectional study; PS, prospective study.*

### *Helicobacter pylori* Stool Antigen Test and Coronary Heart Disease

**FIGURE 6:**
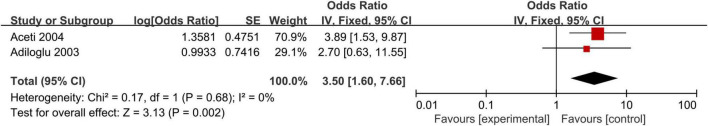
Relationship *H. pylori* stool antigen test and CHD.

We observed a statistically significant association between positive *H. pylori* stool antigen and the development of CHD (OR, 3.50; 95% CI: 1.60–7.66; Figure 6). Because of the lack of data, subgroup meta-analyses and sensitivity analysis could not be conducted for *H. pylori* stool antigen.

### *Helicobacter pylori* Histological Staining Test and Coronary Heart Disease

**FIGURE 7:**
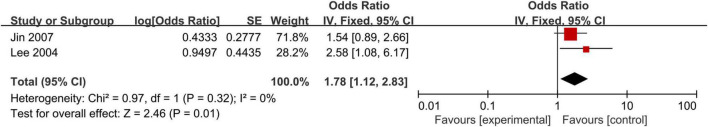
Relationship *H. pylori* histological staining test and CHD.

We also observed that positive *H. pylori* histological staining was significantly associated with the risk of CHD (OR, 1.78; 95% CI: 1.12–2.83; Figure 7). Due to limited data, subgroup meta-analyses and sensitivity analysis of *H. pylori* histological staining also could not be performed.

## Discussion

Over the past 30 years, there has been controversy in the literature about the impact of *H. pylori* infection on the risk of CHD. Therefore, we conducted this updated meta-analysis based on all available studies in order to establish a more comprehensive and stronger analysis. The final results of our meta-analysis showed that the positive anti-*H. pylori* IgG was closely related to the risk of CHD. In addition, this relation was also significant in analysis of positive anti-CagA, positive *H. pylori* stool antigen, and positive *H. pylori* histological staining.

Earlier studies showed that there may be a weak association between *H. pylori* infection and the risk of CHD (64, 65). Currently, our results are similar to those of recent meta-analyses (4, 15, 66–68) in which positive anti-*H. pylori* IgG was positively related to the risk of CHD. However, our study avoided the main limitations of these meta-analyses. For example, the meta-analyses performed by Wang et al. (4), Liu et al. (66), and Rahmani et al. (67) only involved myocardial infarction and did not mention other types of CHD. A meta-analysis (67) conducted in 2017 showed that *H. pylori* infection was associated with an increased risk of CHD, but the meta-analysis was based on Iranians and the number of studies was limited. In addition, a meta-analysis by Sun et al. (15), published in 2016, was based only on prospective studies, excluding cross-sectional studies with stronger evidence. Therefore, the sample size of the included studies was small, which makes the results more prone to confounding factors and selection bias. Similarly, our results are consistent with a previous meta-analysis that indicated a significant association between positive anti-CagA and the risk of CHD. However, our meta-analysis avoided many defects of previous studies (68, 69). For instance, a meta-analysis conducted by Zhang et al. (68) was based only on cross-sectional studies, excluding more meaningful prospective studies. A meta-analysis (69), published in 2006, had a limited number of studies included and had no subgroup analysis for finding the source of heterogeneity. In addition, we also found that positive *H. pylori* stool antigen and positive *H. pylori* histological staining were significantly associated with the risk of CHD. Subgroup analysis and sensitivity analysis were not performed due to the small number of studies.

The mechanism of *H. pylori* infection causing CHD mainly consists of the following aspects. *H. pylori* in atherosclerotic plaque can stimulate inflammatory cells and cause excessive production of cytokines, which leads to local endothelial and vascular dysfunction (30). *H. pylori* infection leads to non-specific stimulation of inflammatory mediators *in vivo*, such as interleukin-1 (IL-1), interleukin-6 (IL-6), C-reactive protein (CRP), and tumor necrosis factor alpha (TNF-α), which promote plaque instability (70). Lastly, it can not only enter endothelial cells through CagA containing exosomes, resulting in endothelial damage (71) but also secrete another virulence factor, vacuolating cytotoxin A (VacA), which can reduce nitric oxide (NO), resulting in endothelial function damage (72).

The expression of P-selectin increases after *H. pylori* infection, and the adhesion between von Willebrand factor (vWF) released by platelets and P-selectin eventually leads to platelet aggregation (73). In addition to this, *H. pylori* infection can affect the risk factors for CHD, such as hypertension, dyslipidemia, hyperhomocysteinemia, diabetes, and impaired glucose tolerance. A recent meta-analysis (74) showed that *H. pylori* infection was significantly associated with arterial hypertension. Aslan et al. (75) found that the levels of total cholesterol, triglyceride, and low-density lipoprotein cholesterol in patients infected with *H. pylori* increased significantly, while high-density lipoprotein cholesterol decreased significantly. The interaction between *H. pylori* infection and diabetes leads to the occurrence of CHD (76). After persistent infection with *H. pylori*, low levels of serum vitamin B12 and folic acid will lead to hyperhomocysteinemia (77).

From our perspective, our meta-analysis is by far the most comprehensive and largest study supported by its statistical ability, leading to a more reliable overall evaluation. Our meta-analysis proved the positive relationship between positive anti-*H. pylori* IgG and development of CHD by subgroup analyses based on setting, category of CHD, adjustment status, and quality assessment score, but this relationship did not appear in prospective studies and some countries. Similarly, we also observed a positive association between the positive anti-CagA and the risk of CHD based on subgroup analyses of adjustment status and quality assessment score, but this association did not exist in prospective studies, community, MI, and Germany. Our results are similar to a previous meta-analysis (15) based on prospective studies, which indicated that *H. pylori* infection increased CHD risk, but this relationship weakens over time. The development of CHD is a multi-effect, long-term process. Over time, other CHD risk factors may attenuate the risk of CHD from the infection. Reasons for other subgroups without *H. pylori* infection increasing the risk of CHD might be associated with fewer studies. In addition, except for the fixed model for studies on anti-*H. pylori* IgG studies reporting HR, *H. pylori* stool antigen, and *H. pylori* histological staining, there is substantial heterogeneity among other studies, which may be attributed to the different participants and different study designs. Hence, the random model is adopted.

### Strengths and Limitations

This is the first attempt to use a meta-analysis to evaluate the relationship between the risk of CHD and *H. pylori* infection through different *H. pylori* detection methods. Most previous meta-analyses used the anti-*H. pylori* IgG test to detect bacterial infection. In fact, this approach fails to detect current infections and may overestimate the association between bacteria and the risk of CHD. However, *H. pylori* stool antigen and *H. pylori* histological staining tests can detect current infection, which accurately assess the relationship between bacteria and the risk of CHD. Since positive anti-CagA has a strong inflammatory response, we also analyzed its association with the risk of CHD. More importantly, the included studies did not adequately consider traditional risk factors for CHD and other microbial infections. In the future, we strongly recommend conducting more well-designed intervention trials and investigating the relationship between other sources of infection and the risk of CHD.

## Conclusion

This meta-analysis revealed an evidence-based relationship between *H. pylori* infection and the risk of CHD, which may contribute to the arguments established in the literature to provide strong evidence. Therefore, we suggest that *H. pylori* infection should be regarded as a new risk factor for CHD in future guidelines related to CHD. Future clinical application needs to be further studied to determine whether early eradication of *H. pylori* can reduce the incidence of CHD.

## Data Availability Statement

The original contributions presented in the study are included in the article/supplementary material, further inquiries can be directed to the corresponding author/s.

## Author Contributions

X-JD and LT designed and analyzed the meta-analysis, and contributed to the revision of the manuscript. X-JD, LT, B-BW, F-HL, S-PL, and F-FP collected the data. All authors have read and approved the manuscript.

## Conflict of Interest

The authors declare that the research was conducted in the absence of any commercial or financial relationships that could be construed as a potential conflict of interest.

## Publisher’s Note

All claims expressed in this article are solely those of the authors and do not necessarily represent those of their affiliated organizations, or those of the publisher, the editors and the reviewers. Any product that may be evaluated in this article, or claim that may be made by its manufacturer, is not guaranteed or endorsed by the publisher.
